# Multivariate data analysis of sex differences in emotional and cognitive evaluations over 1 year after stroke

**DOI:** 10.1038/s41598-025-32534-5

**Published:** 2025-12-19

**Authors:** Suhrit Duttagupta, Thomas Tourdias, Sharmila Sagnier, Mathilde Poli, Sabrina Debruxelles, Pauline Renou, Stéphane Olindo, Igor Sibon, Sylvie Berthoz

**Affiliations:** 1 University of Bordeaux, INCIA UMR 5287, Bordeaux, France; 2 University of Bordeaux, Neurocentre Magendie UMR 1215, Bordeaux, France; 3 CHU Bordeaux, Diagnostic and Therapeutic Neuroimaging Unit, Bordeaux, France; 4 CHU Bordeaux, Stroke Unit, Bordeaux, France; 5 Department of Psychiatry for Adolescents and Young Adults, Institut Mutualiste Montsouris, Paris, France

**Keywords:** Stroke, Sex differences, Cognitive impairments, Depression, Anxiety, Quality of life, Diseases, Health care, Medical research, Neurology, Neuroscience

## Abstract

Post-stroke disabilities in cognition and mood lead to worse stroke recovery trajectory but are frequently overlooked. Although neurological factors and clinical history have been documented as important predictors of these invisible handicaps, the role of sex has not been given enough scrutiny. Examining sex-based differences in these outcomes could help deliver better post-stroke care. The goal of this study was to explore the interplay over one year between post-stroke cognitive and socio-affective assessments for men and women separately. Clinical evaluations of a monocentric hospital-based cohort including 263 patients with first-ever ischemic stroke were taken before hospital discharge and at 3- and 12-months post-stroke. Univariate comparisons between men and women were conducted, followed by multivariate analyses controlling for stroke severity, age, and education. Partial correlations between neuroradiological (stroke volume, white matter hyperintensities), cognitive (Montreal Cognitive Assessment test), mood (Hospital Anxiety and Depression scale; Apathy Inventory), and quality-of-life (Life Satisfaction Questionnaire-9) metrics were computed for both sexes. In multivariate analyses, women showed higher levels of baseline depression (p_adj_ < 0.05) and apathy Initiate (p_adj_ < 0.05) and Interest (p_adj_ < 0.001) subscores, as well as of anxiety at both follow-ups (p_adj_ < 0.05); they also endorsed lower scores in various quality-of-life sub-domains across all time points. Men had increasing levels of depression over time and showed stronger associations between psychological outcomes, including greater intercorrelations between cognitive assessments. Only spurious associations were found between clinical and neuroradiological characteristics for both sexes. Independently from stroke severity, age, and education, there were notable sex differences in the interplay between post-stroke cognitive and socio-affective functioning, suggesting differences in resilience and resistance to pathological burden. The inclusion of sex- and gender-specific factors in clinical evaluations seems critical to optimize post-stroke care strategies.

*Clinical Trial Registration*: Trial name: Brain Before Stroke; ID: PHRC-12-152; URL: https://scanr.enseignementsup-recherche.gouv.fr/project/PHRC-12-152.

## Introduction

Stroke is one of the leading causes of death and disability worldwide. Apart from the motor impairments commonly associated with stroke, disabilities in psychological functioning may contribute to severe restricted participation in daily life activities and poor quality of life (QoL)^1–3^. Post-stroke cognitive impairments (PSCI) and depression (PSD), which are respectively observed in 50% and 33% of stroke survivors, are the most frequent forms of invisible post-stroke handicaps^4,5^. Moreover, post-stroke anxiety (PSA) and apathy are two additional frequent complaints (22% and 41% respectively), which may co-exist with PSCI and/or PSD or occur independently^6^. Since these conditions lead to worse stroke outcomes, they require optimal management.

Apart from antecedents of cognitive or mood problems, clinical severity, and neuroradiological markers such as stroke volume and extent of white matter hyperintensities (WMH), few predictors contributing to the poor psychological status of stroke patients have been consistently identified across studies^7,8^.

Among the various demographic factors likely to influence post-stroke psychological outcomes and the interactions between cognitive and emotional manifestations, the role of sex has surprisingly received little attention. In recent years, a growing number of studies have highlighted significant differences in the stroke characteristics of women compared with men, such as delayed diagnosis, older age at stroke onset, greater severity, less access to reperfusion strategies, different aetiological mechanisms with a greater frequency of atrial fibrillation and a poorer functional prognosis^9,10^. Nevertheless, after adjusting for confounding variables, some data point to a more favourable prognosis in women, suggesting that mechanisms of adaptation and cerebral resilience may be different according to sex^11^.

The exploration of sex-specific post-stroke cognitive and emotional outcomes has been limited. This is primarily due to sex being taken as a confounding variable while conducting analysis on clinical or neuroimaging data^12^, due to which the effect of sex on clinical evaluations is not reported as a variable of interest. Up to now, a systematic review found mixed results for sex-differences of PSCI^13^. The most recent meta-analysis on nonmotor post-stroke adverse outcomes found that female sex was significantly associated with depression and sleep disturbances but not anxiety, fatigue, pain and social participation^14^. While another meta-analysis found no difference in sexes for QoL and PSD, this was based on two and three studies, respectively^15^. No meta-analyses evaluating sex differences in PSA or post-stroke apathy are available. Furthermore, the associations between psychological and neurological variables, as well as the interactions between cognitive and emotional manifestations, specifically within each sex remain overlooked, underscoring the need for examining sex-specific interplay between these variables.

This study aimed to gain knowledge in this domain and help guide clinical practice by identifying sex-based differences in post-stroke cognitive and socio-affective status over one year, and exploring potential links between these outcomes within sexes.

## Methods

### Study procedure

Participants were part of the “Brain Before Stroke” protocol, a longitudinal monocentric research protocol (Bordeaux University Hospital, France). Patients included were adults having a first-ever ischemic stroke within 24–72 h and not showing prestroke disability. Detailed description of inclusion criteria and MRI acquisition have been previously published^16^. The protocol was validated by the regional ethics committee (2012/19 2012-A00190-43). The methods were conducted in accordance with the Declaration of Helsinki and the Institutional Review Boards and local ethics committee (CPP Sud-Est III) approved the study protocol. A written informed consent was provided by all participants.

### Clinical evaluation

Demographic data and risk factors were recorded at baseline. Education was recorded based on the French system of education levels. Pre-stroke cognition was evaluated retrospectively after stroke onset—within two days of admission, in accordance with the guidelines—based on the responses of a relative using the Informant Questionnaire in Cognitive Decline in the Elderly (IQCODE)^17^. Post-stroke evaluations were performed before hospital discharge (M0), and after three (M3) and 12 months (M12), and included the National Institutes of Health Stroke Scale (NIHSS), the modified Rankin Scale (mRS)^18^, the Montreal Cognitive Assessment (MoCA)^19^, the Hospital Anxiety and Depression (HAD) scale^20^, the Apathy Inventory (AI)^21^, and the Life Satisfaction Questionnaire 9 (LISAT-9)^22^. At M0, the LISAT-9 evaluated pre-stroke QoL, the HAD evaluated mood over the past week, and the AI for the past fortnight.

### MRI analysis

Masks of the ischemic stroke lesion and WMH were created with the software 3D Slicer, version 4.3.1 (https://www.slicer.org/). The infarct and WMH were delineated using the Lesion Segmentation Tool^23^ and their volumes were calculated using 3D Slicer. WMH was used as a separate marker because it depicts brain frailty and has been associated with post-stroke nonmotor adverse outcomes^24,25^.

### Statistical analysis

Data were only analysed for patients with no missing evaluations of interest. All analyses were conducted on R^26^. Based on the cutoff score of 7 for both anxiety and depression from the HAD, the proportion and incidence of potential mood disorder by sex were calculated. Sex-based differences were determined by Mann–Whitney for quantitative variables and by χ^2^ or Fisher’s exact tests for qualitative variables. To compare sexes for evaluations with significant univariate difference, Quade tests were performed while controlling for NIHSS score, age, and education. Quade is a ranked-based non-parametric alternative to ANCOVA; instead of the raw scores used for ANCOVA, the residuals are ranked after adjusting for covariates and group differences are tested using these residuals. Spearman correlation matrices were computed between the variables of interest for each sex, taking significant correlations after false discovery rate (FDR) correction. Partial correlations were performed between all variables while controlling for NIHSS score at each timepoint, age, and education. The IQCODE and pre-stroke mRS were not included in the analyses due to low variance resulting from the exclusion of subjects with severe disability. To examine the interaction between sex and time of assessment, analysis of variance (ANOVA) tests were performed with the Aligned Rank Transform of the variables.

Out of the 263 (Men/Women: 178/85) participants included at baseline, and using ages under 55 for the classification of young ischemic stroke^14^ the distribution was 205 older stroke patients (Men/Women: 136/69) and 58 younger ones (Men/Women: 42/16). At follow-up, 236 patients were present at M3 (Men/Women: 160/76) and 182 at M12 (Men/Women: 127/55). The only significant differences were lower MoCA Language and lower LISAT-9 Functionality scores (both *p* < 0.05) among the patients lost to follow-up than the overall inclusion sample.

## Results

The main demographic and clinical characteristics of the overall population at M0 are reported in Table 1. The sex-based univariate comparisons for all time-points are described in Table 2. Based on univariate between-sex comparisons, women were significantly older (mean difference 5.5 years; *p* < 0.001), had lower education (*p* = 0.037) as well as lower rates of alcohol and tobacco consumption (*p* < 0.001). There was no significant association between alcohol or tobacco use and the proportion of patients with clinically-significant anxiety or depression at M0.

**Table 1:** Descriptive statistics and between-sex comparisons at baseline.

	Men (n = 178)	Women (n = 85)	Statistic (*p* value)
Age, mean (SD)	63.9 (12.4)	**69.4 (16.0)**	5457 (< 0.001)
Right-handed, N (%)	165 (95)	80 (94)	0.02 (0.754)
Education			
None	**3**	3	10.2 (0.037)
Primary	**22**	22	
Junior High School	**69**	26	
High School	**22**	9	
Higher Education	**44**	14	
Vascular Risk Factors			
Atherosclerosis, N (%)	114 (64)	53 (62)	0. 532 (0.792)
Diabetes, N (%)	35 (16)	23 (18)	0.395 (0.531)
Atrial Fibrillation, N (%)	26 (12)	14 (16)	0.155 (0.694)
Tobacco use, N (%)	**104 (58)**	22 (27)	24.9 (< 0.001)
Alcohol use, N (%)	**48 (27)**	8 (9)	10.6 (< 0.001)
Hypercholesterolemia, N (%)	85 (48)	35 (41)	0.852 (0.360)
Hypertension, N (%)	104 (58)	47 (54)	0.071 (0.790)
Obesity, N (%)	30 (17)	20 (24)	0.901 (0.196)
Pre-stroke Status			
IQCODE, median (IQR)	3 (0)	3 (0)	0.2
Neuroanatomical Status			
Stroke Volume (mm^3^), mean (SD)	7.09 (31.5)	11.0 (35.6)	6383 (0.384)
WMH Volume (mm^3^), mean (SD)	17 (15.5)	16.3 (32.0)	6750 (0.856)

n = number of cases; %: percentage; SD: Standard Deviation; IQR: interquartile range; Bold cells show which sex had higher values.

**Table 2:** Non-parametric between-sex comparisons for variables of interest.

	Overall median (IQR)	Men median (IQR)	Women median (IQR)	Statistic (*p* value)
NIHSS				
M0	3 (4)	3 (4)	3 (5)	6737 (0.146)
M3	1 (2)	1 (2)	1 (3)	5393 (0.164)
M12	0.5 (2)	0 (1)	**1 (2.5)**	2817 (0.026)
mRS				
M0	0 (0)	0 (0)	0 (0)	5798 (0.072)
M3	1 (2)	1 (2)	1 (2)	4170 (0.151)
M12	1 (2)	1 (2)	**1 (3)**	2751 (0.019) [2.13 (p_adj_ = 0.140)]
HAD Anxiety				
M0	6 (6)	6 (5)	**7 (7)**	6141 (0.013) [4.66 (p_adj_ = 0.081)]
M3	6 (5)	5 (4)	**7 (6)**	4135 (< 0.001) [16.8 (p_adj_ < 0.001)]
M12	5 (5)	5 (5)	**7 (6)**	2743 (0.021) [4.26 (p_adj_ = 0.040)]
HAD Depression				
M0	3 (4)	2 (4)	**4 (5)**	5696 (0.001) [5.3 (p_adj_ = 0.048)]
M3	3 (5)	3 (5)	3 (4)	5388 (0.181)
M12	3 (6)	3 (5)	4 (7)	3115 (0.245)
LISAT-9 Total				
M0	43 (13)	**44 (7)**	41 (13)	5413 (0.008) [6.5 (p_adj_ = 0.049)]
M3	42 (9.25)	**43 (9)**	40 (9)	4349 (< 0.001) [10.6 (p_adj_ < 0.001)]
M12	42 (10.8)	**42 (9)**	38 (13)	2423 (0.002) [2.7 (p_adj_ = 0.104)]
LISAT-9 Life				
M0	5 (2)	**5 (2)**	5 (2)	6094 (0.006) [6.52 (p_adj_ = 0.011)]
M3	5 (1)	**5 (1)**	4 (1)	4689 (0.004) [14.87 (p_adj_ = 0.007)]
M12	5 (1)	5 (1)	5 (1)	3049 (0.148)
LISAT-9 Functionality				
M0	5 (2)	5 (2)	5 (2)	7489 (0.993)
M3	5 (1)	6 (1)	5 (2)	4740 (0.004) [5.06 (p_adj_ = 0.025)]
M12	6 (1)	6 (1)	5 (3)	2683 (0.007) [1.48 (p_adj_ = 0.225)]
LISAT-9 Hobbies				
M0	5 (1)	5 (1)	5 (2)	7018 (0.337)
M3	5 (1)	**5 (1.25)**	4 (2)	4943 (0.021) [5.90 (p_adj_ = 0.022)]
M12	5 (1)	**5 (1)**	4 (2)	2605 (0.005) [13.2 (p_adj_ = 0.128)]
LISAT-9 Profession				
M0	5 (2)	5 (2)	5 (2)	6833 (0.108)
M3	5 (2)	5 (2)	5 (3)	5647 (0.407)
M12	5 (3)	**5 (2)**	4 (3)	2856 (0.045) [1.94 (p_adj_ = 0.165)]
LISAT-9 Finance				
M0	5 (1)	5 (1)	5 (1)	7053 (0.294)
M3	5 (1)	5 (1)	4 (2)	5532 (0.286)
M12	5 (1)	5 (1)	4 (1)	3120 (0.148)
LISAT-9 Sex				
M0	4 (2)	**5 (2)**	4 (3)	6071 (0.010) [2.89 (p_adj_ = 0.071)]
M3	4 (2)	**4 (2)**	3 (4)	4915 (0.013) [4.61 (p_adj_ = 0.04)]
M12	4 (3)	**4 (3)**	3 (3)	2413 (0.008) [1.37 (p_adj_ = 0.286)]
LISAT-9 Partner				
M0	5 (2)	**5 (2)**	5 (3)	5836 (0.003) [5.67 (p_adj_ = 0.018)]
M3	5 (2)	**5 (2)**	4 (3)	4210 (< 0.001) [15.2 (p_adj_ < 0.001)]
M12	5 (3)	**5 (2)**	3 (2.5)	2155 (< 0.001) [6.44 (p_adj_ = 0.016)]
LISAT-9 Family				
M0	5 (1)	5 (1)	5 (2)	6841 (0.206)
M3	5 (1)	5 (1)	5 (1)	5931 (0.816)
M12	5 (1)	5 (1)	5 (1)	3415 (0.641)
LISAT-9 Friends				
M0	5 (1)	5 (1)	5 (1)	7121 (0.544)
M3	5 (1)	5 (1)	5 (1)	5974 (0.889)
M12	5 (2)	5 (2)	5 (1)	3313 (0.474)
Apathy Total				
M0	0 (0)	0 (0)	**0 (3)**	6733 (0.045) [3.05 (p_adj_ = 0.082)]
M3	0 (0)	0 (0)	0 (0)	5734 (0.512)
M12	0 (6)	0 (6.5)	0 (8)	3673 (0.648)
Apathy Affect				
M0	0 (0)	0 (0)	0 (0)	7247 (0.271)
M3	0 (0)	0 (0)	0 (0)	6016 (0.945)
M12	0 (0)	0 (2)	0 (0)	0.676
Apathy Initiate				
M0	0 (0)	0 (2)	**0 (2)**	6607 (< 0.001) [4.1 (p_adj_ = 0.026)]
M3	0 (0)	0 (0)	0 (2.5)	5667 (0.327)
M12	0 (2)	0 (2)	0 (3.5)	3325 (0.730)
Apathy Interest				
M0	0 (0)	0 (2)	**0 (2)**	6664 (0.005) [16.5 (p_adj_ < 0.001)]
M3	0 (0)	0 (0)	0 (0)	5849 (0.563)
M12	0 (0)	0 (1.5)	0 (0)	2795 (0.726)
MoCA Total				
M0	23 (8)	**24 (8.75)**	22 (8)	6899 (0.042) [0.74 (p_adj_ = 0.744)]
M3	25 (6)	**26 (5)**	24 (6)	4710 (0.008) [0.298 (p_adj_ = 0.586)]
M12	26 (5)	26 (4)	25 (5)	3291 (0.443)
MoCA Visuospatial				
M0	3 (3)	**4 (3.75)**	3 (3)	5179 (0.002) [6.24 (p_adj_ = 0.016)]
M3	4 (2)	**4 (2)**	3 (2)	4830 (0.008) [1.98 (p_adj_ = 0.161)]
M12	4 (2)	**4 (1)**	4 (2)	2542 (0.002) [10.73 (p_adj_ = 0.013)]
MoCA Denomination				
M0	3 (0)	**3 (0)**	3 (1)	5682 (0.020) [7.49 (p_adj_ = 0.015)]
M3	3 (0)	3 (0)	3 (0)	5522 (0.104)
M12	3 (0)	3 (0)	3 (0)	3266 (0.181)
MoCA Attention				
M0	5 (2)	**5 (2)**	5 (3)	6242 (0.011) [6.12 (p_adj_ = 0.049)]
M3	5 (1)	5 (1)	5 (2)	5177 (0.058)
M12	6 (1)	6 (1)	6 (1.5)	3343 (0.615)
MoCA Language				
M0	2 (1)	**2 (2)**	2 (1)	6929 (0.026) [1.12 (p_adj_ = 0.480)]
M3	2 (1)	2 (1)	2 (1)	5357 (0.119)
M12	3 (1)	3 (1)	2 (1)	3222 (0.347)
MoCA Abstract				
M0	2 (1)	2 (1)	2 (1)	7612 (0.397)
M3	2 (1)	**2 (1)**	1 (1)	4738 (0.003) [4.28 (p_adj_ = 0.095)]
M12	2 (1)	**2 (1)**	2 (1)	2862 (0.020) [1.23 (p_adj_ = 0.148)]
MoCA Memory				
M0	2 (4)	2 (4)	3 (3)	6805 (0.407)
M3	3 (3)	3 (3)	3 (3)	5635 (0.400)
M12	4 (3)	3 (2)	**4 (2)**	2991 (0.05) [6.60 (p_adj_ = 0.011)]
MoCA Orientation				
M0	6 (1)	6 (0.75)	6 (1)	7991 (0.472)
M3	6 (0)	6 (0)	6 (0)	5778 (0.379)
M12	6 (0)	6 (0)	6 (0)	3260 (0.209)

Between-sex comparisons for clinical evaluations at baseline (M0), 3 months (M3), and 12 months (M12) post-stroke. For the variables with significant differences, Quade analyses (in square brackets, p_adj_: *p* value of the Quade analysis) were performed taking age, National Institutes of Stroke Scale (NIHSS), and education as covariates; mRS: modified Rankin Scale; HAD: Hospital Anxiety and Depression; LISAT-9 : Life Satisfaction Questionnaire 9; MoCA: Montreal Cognitive Assessment; n = number of cases; IQR: interquartile range; Bold cells indicate the sex with significantly higher values.

At M0, women had significantly: higher HAD anxiety (*p* = 0.013) and depression (*p* < 0.001), as well as Apathy Initiate (*p* < 0.001) and Interest subscores (*p* < 0.005); lower LISAT-9 Total, Life, Sex and Partner scores (*p* < 0.01); lower MoCA Total, Visuospatial, Denomination, Attention and Language scores (*p* < 0.05). At M3 and M12, women still endorsed significantly higher HAD anxiety scores (M3: *p* < 0.001; M12: *p* = 0.02), had significantly lower LISAT-9 Total, Functionality, Sex and Partner scores (*p* < 0.05), as well as lower MoCA Abstraction (*p* < 0.05), Visuospatial scores (*p* < 0.01), and Memory (only at M12; *p* = 0.05).

Using multivariate Quade analyses, the sex differences remained significant for HAD Depression at M0 (95% CI, − 0.021 to 0.014; p_adj_ < 0.05) and for HAD Anxiety at both follow-ups (p_adj_ < 0.05), for apathy Initiate and Interest at M0 (p_adj_ < 0.05; p_adj_ < 0.001). For the sex differences in the LISAT-9, Total and Life remained significant at M0 (p_adj_ < 0.05) and M3 (p_adj_ < 0.01), Functionality, Hobbies, and Sex remained significant at M3 (all p_adj_ < 0.05), while Partner was significant at all timepoints (*p* < 0.05). For the MoCA, the only sex differences that remained significant were Attention, Denomination, and Visuospatial at M0 (p_adj_ < 0.05), and Visuospatial and Memory at M12 (p_adj_ < 0.05).

**Fig. 1:**
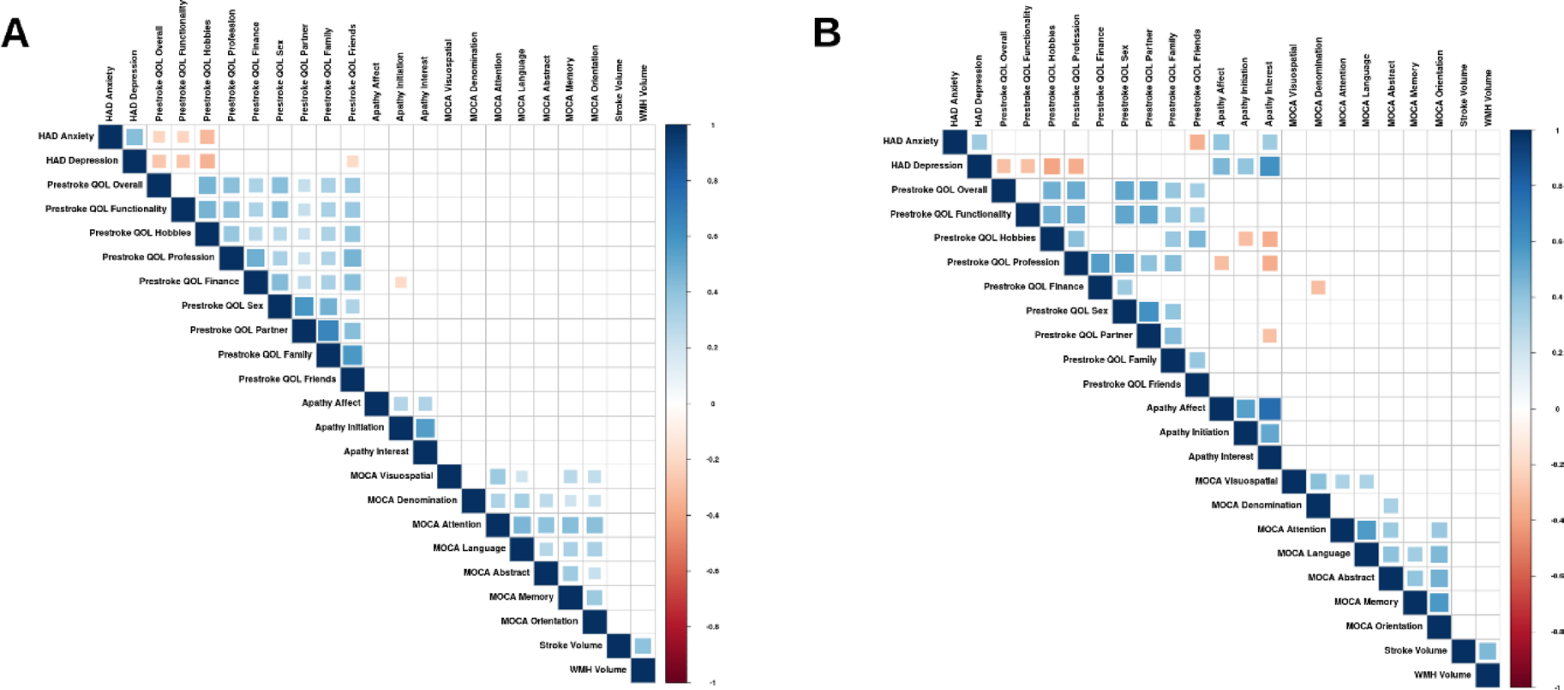
Correlograms for post-stroke evaluations at baseline. Partial non-parametric correlations between the variables of interest for (**A**) Men and (**B**) Women while controlling for National Institutes of Health Stroke Scale (NIHSS), age, and education at baseline. Larger squares indicate more significant correlations after applying FDR correction. HAD: Hospital Anxiety and Depression; MOCA: Montreal Cognitive Assessment; QOL: Life Satisfaction Questionnaire 9; WMH: White Matter Hyperintensities.

**Fig. 2:**
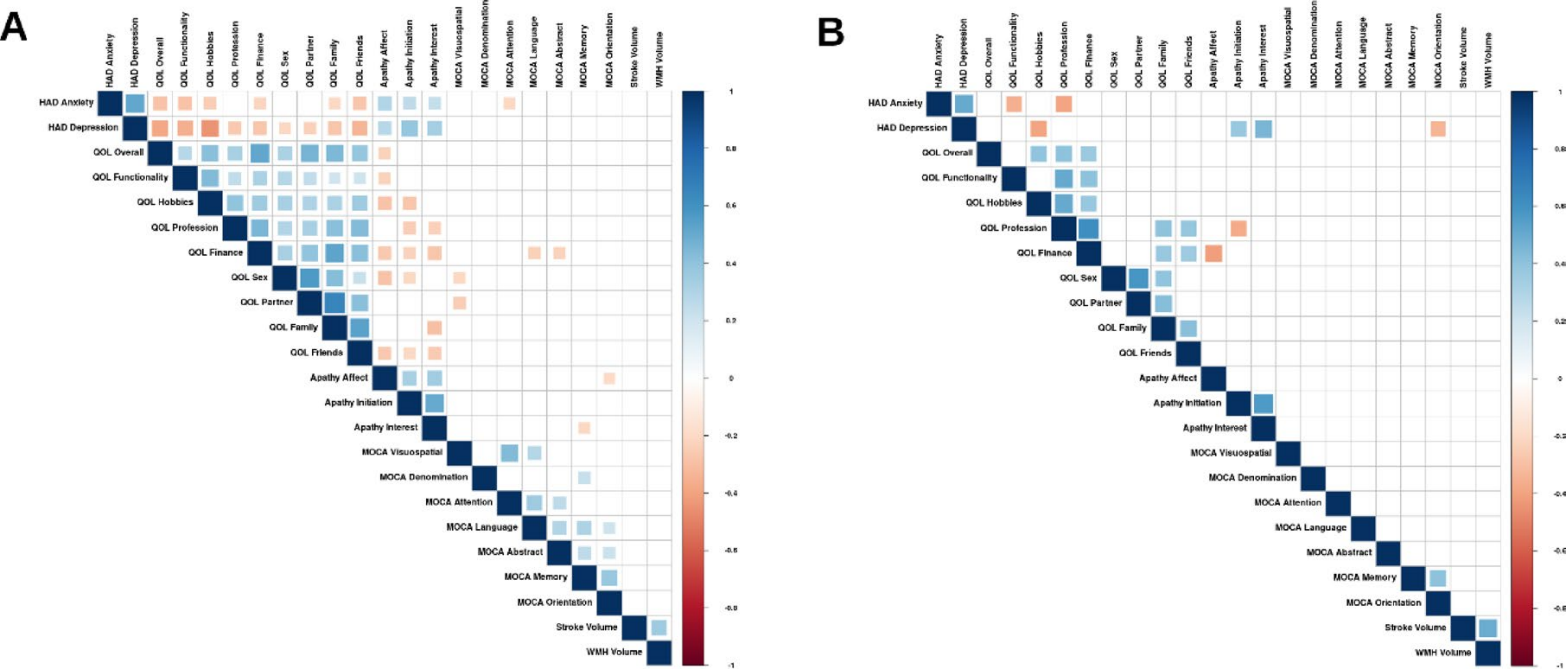
Correlograms for post-stroke evaluations at 3 months. Partial non-parametric correlations between the variables of interest for (**A**) Men and (**B**) Women while controlling for National Institutes of Health Stroke Scale (NIHSS), age, and education at 3 months post-stroke. Larger squares indicate more significant correlations after applying FDR correction. HAD: Hospital Anxiety and Depression; MOCA: Montreal Cognitive Assessment; QOL: Life Satisfaction Questionnaire 9; WMH: White Matter Hyperintensities.

**Fig. 3:**
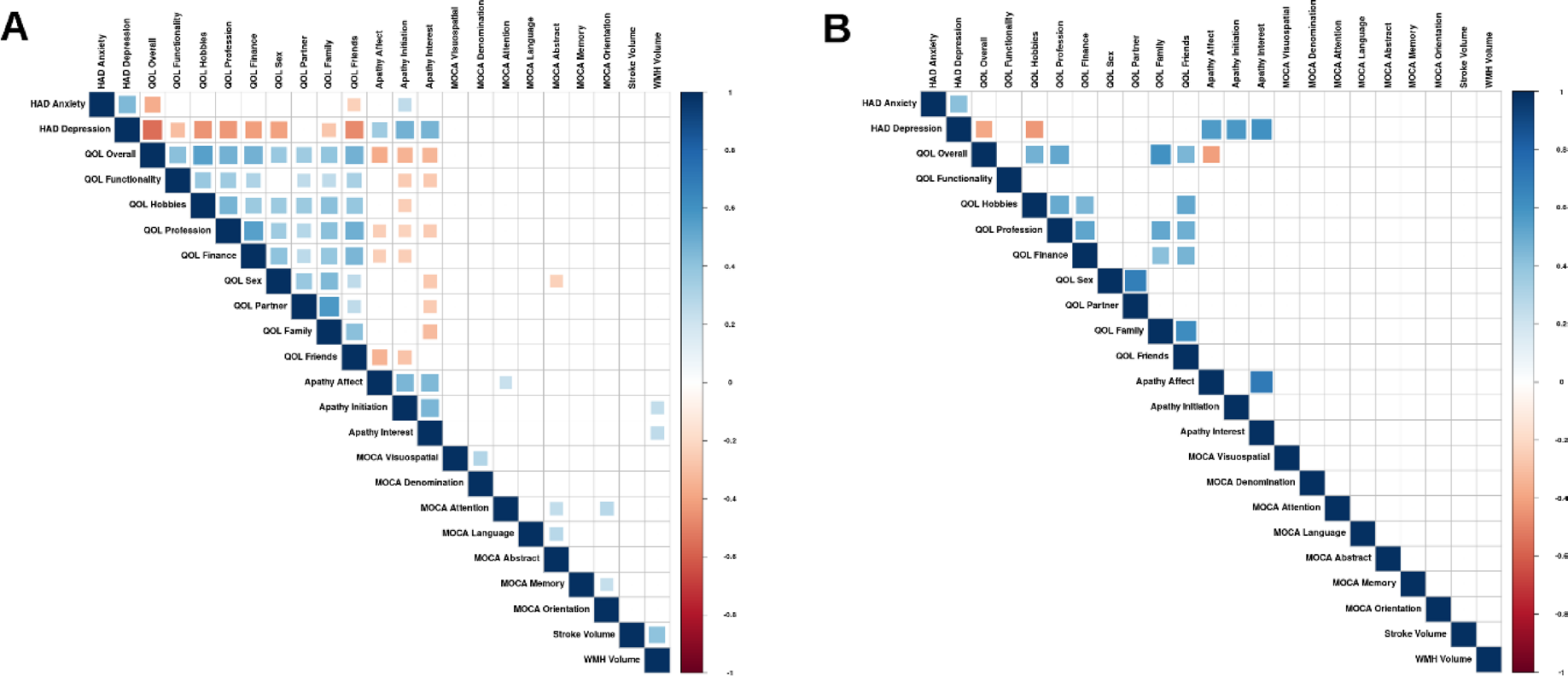
Correlograms for post-stroke evaluations at 1 year. Partial non-parametric correlations between the variables of interest for (**A**) Men and (**B**) Women while controlling for National Institutes of Health Stroke Scale (NIHSS), age, and education at 12 months post-stroke. Larger squares indicate more significant correlations after applying FDR correction. HAD: Hospital Anxiety and Depression; MOCA: Montreal Cognitive Assessment; QOL: Life Satisfaction Questionnaire 9; WMH: White Matter Hyperintensities.

Partial correlations matrices for the variables of interest at all timepoints by sex are shown in Fig. 1 for M0, Fig. 2 for M3, and Fig. 3 for M12. Only significant associations are reported below.

At M0, only men had negative associations between HAD Anxiety and LISAT-9 Overall Life (p_FDRcorr_ < 0.05), Functionality (p_FDRcorr_ < 0.05), and Hobbies (p_FDRcorr_ < 0.001), while both sexes had negative associations between HAD Depression and LISAT-9 Overall Life (p_FDRcorr_ < 0.05), Functionality (p_FDRcorr_ < 0.05), and Hobbies (p_FDRcorr_ < 0.001). Only women had positive associations between Apathy indices and HAD Anxiety and Depression (p_FDRcorr_ < 0.01).

At M3, men had more significant associations between HAD Depression, Anxiety as well as Apathy and LISAT-9 scores (negative; p_FDRcorr_ < 0.001). Only men had a negative association between HAD Anxiety and MoCA Attention (p_FDRcorr_ < 0.05), while only women had a negative association between HAD Depression and MoCA Orientation (p_FDRcorr_ < 0.05).

At M12, men had more negative associations between LISAT-9 scores and HAD Depression (p_FDRcorr_ < 0.001), Anxiety (p_FDRcorr_ < 0.05), as well as apathy indices (p_FDRcorr_ < 0.01). Additionally, only men had associations between MoCA Attention and Apathy Affect (positive, p_FDRcorr_ < 0.05), between MoCA Abstract and LISAT-9 Sex (negative, p_FDRcorr_ < 0.05), between WMH volume and Apathy Initiation (positive, p_FDRcorr_ < 0.05) and Interest (positive, p_FDRcorr_ < 0.05).

The prevalence and incidence of PSA and PSD at each timepoint are listed in Table 3. Based on the clinical thresholds of the HAD scale, the prevalence of clinically-significant anxiety at the three timepoints were 36.5%, 46.7%, and 40.0% respectively among women, while it was 28.7%, 24.8%, and 26.8% respectively among men. The proportions were significantly different between sexes overall (χ^2^ = 23.5, *p* < 0.001) and individually at M0 (χ^2^ = 9.7, *p* = 0.002) and M3 (χ^2^ = 19.2, *p* < 0.001). In the case of clinically-significant depression, the corresponding prevalences were 22.4%, 20.0%, and 27.3% among women, while it was 7.3%, 14.3%, and 18.9% among men. The proportions were significantly different between sexes overall (χ^2^ = 10.76, *p* < 0.001) and at M0 (χ^2^ = 12.19, *p *< 0.001). The incidence of post-stroke anxiety at M3 and M12 in women was 12.7% and 9.2% respectively, while in men it was 10.5% and 5.6% respectively. The incidence of post-stroke depression at M3 and M12 in women was 11.4% and 9.8% respectively, while in men it was 10.5% and 8.1% respectively.

**Table 3:** Prevalence and incidence of post-stroke anxiety and depression.

	Prevalence	(%)	Incidence	(%)
	Male	Female	Male	Female
HAD anxiety				
M0	28.7	36.5	–	–
M3	24.8	46.7	10.5	12.7
M12	26.8	40.0	5.6	9.2
HAD depression				
M0	7.3	22.4	–	–
M3	14.3	20.0	10.5	11.4
M12	18.9	27.3	8.1	9.8

Values determined using the clinical threshold of 8 or above on the HAD Anxiety or Depression subscores. M0: Baseline, M3: 3 months post-stroke; M12: 12 months post-stroke.

**Fig. 4:**
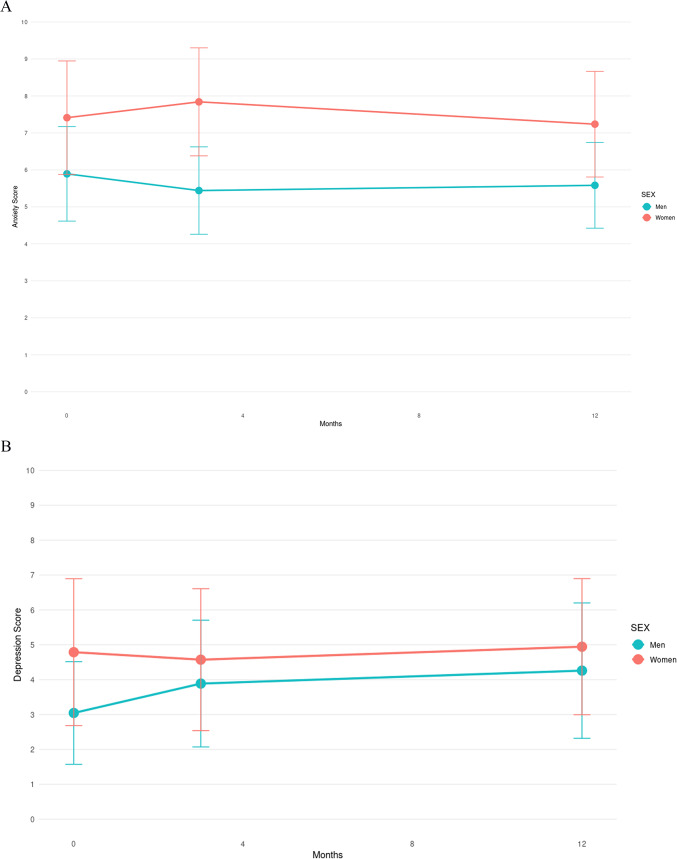

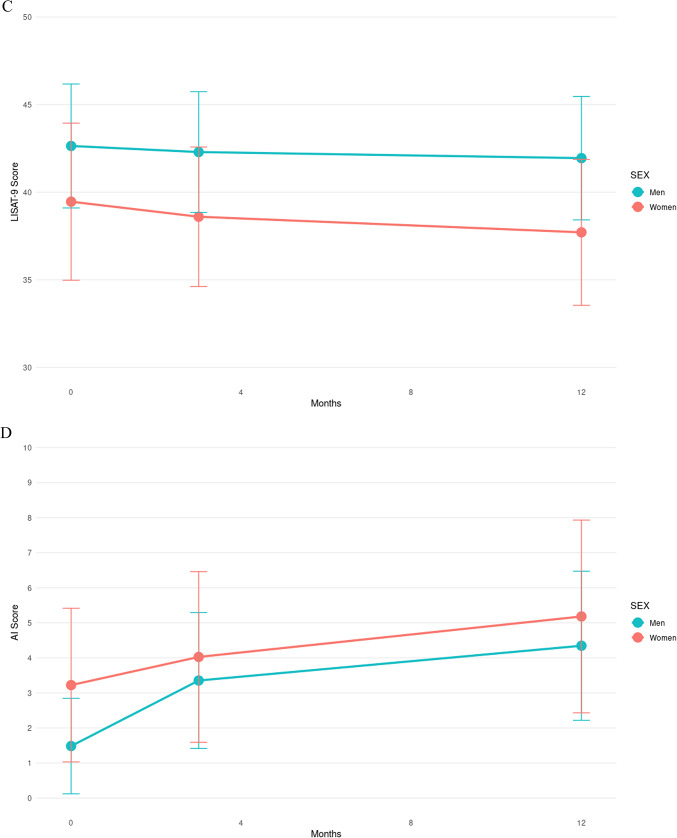

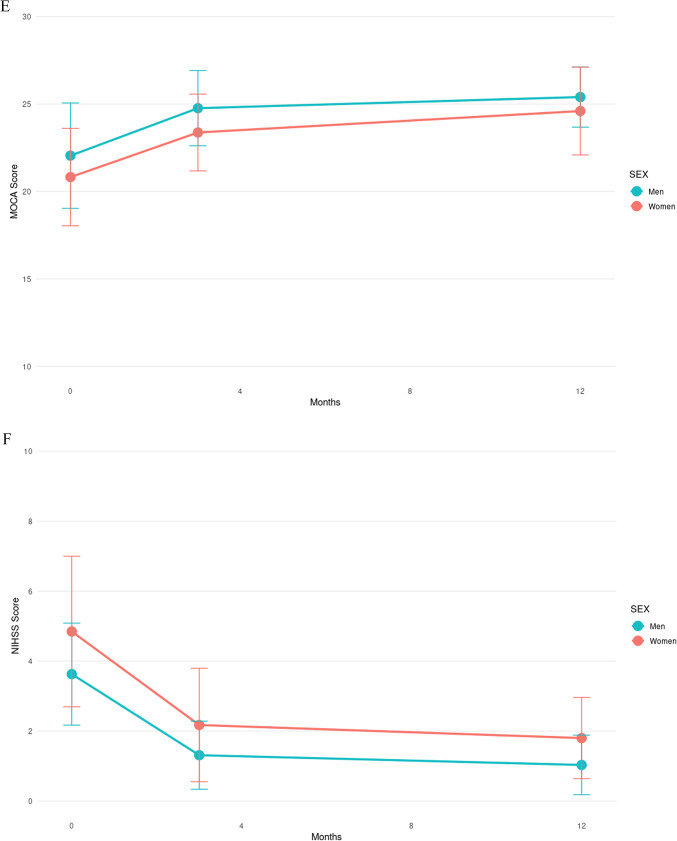
Progression of post-stroke clinical evaluations over time. Progression of scores of (**A**) Hospital Anxiety and Depression (HAD) Anxiety, (**B**) HAD Depression, (**C**) Life Satisfaction Questionnaire 9 (LISAT-9), (**D**) Apathy Index (AI), (**E**) Montreal Cognitive Assessment (MoCA), and (**F**) National Institutes of Health Stroke Scale scores for men (blue) and women (red) with the mean score and standard deviation at each timepoint of examination.

Figure 4 shows the progression of clinical evaluation scores by sex. Through ranked ANOVA tests, only the NIHSS score had a significant interaction between the time of evaluation and sex (*p* < 0.001). None of the proportions were significantly different between sexes.

## Discussion

The main findings of the present study, which investigated potential differences in cognitive and socio-affective functioning between men and women stroke survivors over one year, are that: (i) in the acute stage, women have a more severe global clinical presentation than men, (ii) there was an increase in depressive mood over time in men only, and (iii) the interplay between post-stroke emotional distress and quality-of-life was stronger among men.

As confirmed in a recent systematic review and meta-analysis, neuropsychiatric difficulties are frequent invisible, adverse nonmotor stroke outcomes that persist over time^14^. While this meta-analysis reported an overall prevalence of 25.8% (95% CI 23.8–27.8%) for PSD and of 26.9% (95% CI 23.7–30.2%) for PSA, sub-analyses showed that study design, age and sex were differentially associated with these conditions: age (binary coded using < 54 years of age versus 55 or higher) was not associated with these two neuropsychiatric conditions (PSA: OR = 0.98, 95% CI 0.71–1.05, *p* = 0.133; PSD: OR = 0.40, 95% CI 0.18–1.05, *p* = 0.120), while a hospital-based (versus Population) design was associated with both PSD (OR = 1.60, 95% CI 1.11–1.82, *p* = 0.008) and PSA (OR = 1.73, 95% CI 1.10–2.17, *p* = 0.017), and female sex was associated with PSD only (PSD: OR = 1.82, 95%CI 1.16–3.97, *p* = 0.034; PSA: OR = 1.11, 95% CI 0.93–1.35, *p* = 0.945).

Independently from age, level of education and stroke severity, at the time of stroke, women endorsed higher levels of emotional distress than men. This aligns with data concerning the most prominent pre-stroke risk factors for PSD^27^ as well as epidemiological data from the general population, with women being nearly twice as likely as men to have an anxiety and/or mood disorder^28,29^. Biological and social factors, such as hormonal fluctuations as well as caregiving and/or domestic responsibilities and/or exposure to domestic violence, contribute to this increased prevalence. Despite this risk of pre-stroke mood disruptions and differences in cognitive function, there was weak interplay between quality of life and cognitive and mood variables in women. This suggests that mood and cognitive disruptions in women exert a lesser impact on their perceived daily status, aligning with research indicating women form stronger adaptive strategies compared to men in managing mood-related impairments in daily life^30,31^.

While levels of anxiety remained more pronounced in women during the one-year follow-up, depression severity remained stable in women but increased in men. Men, who are known to prioritize physical independence and productivity, may experience intensified feelings of inadequacy and depression when faced with stroke-induced disabilities^32^. Conversely, women have been shown to have broader social support networks and are more inclined to seek emotional assistance, which could mollify depressive mood^33,34^. Although there is certain evidence in the literature to justify this observation, only broad explanations can be posited because of insufficient documentation. Future studies focusing specifically on this aspect will be essential to clarify this, particularly through qualitative analyses.

Over the course of the study, stronger associations between depression, and to a lesser extent anxiety, and quality of life were observed in men compared to women at follow-up. Additionally, the interplay between quality-of-life sub-domains were more pronounced in men. Men tend to exhibit a narrower range of interests compared to women, with a predominant focus on physical activities^35,36^. In contrast, women often demonstrate a broader diversity of interests spanning social, cultural, and cognitive domains. This disparity in interest diversity may influence the psychological and social resilience of men and women in response to life-altering events such as stroke. For men, the reduced participation in work or social engagements impairing quality of life could be driving stronger correlations between mood impairments and quality-of-life factors^37^.

Sex differences were also observed based on apathy, as men exclusively showed correlations between apathy and quality of life metrics as well as between apathy and anxiety. Mood disruptions are frequently accompanied by apathy, a condition characterized by reduced motivation, diminished goal-directed behaviors, and emotional indifference. Although apathy often coexists with post-stroke depression, it can also occur independently in up to 40% of cases^38^. To the best of our knowledge, no study in stroke populations has examined the correlations between apathy and mood separately for sexes. However, there were similar results from studies examining sex differences for sub-dimensions of apathy in patients suffering from HIV/AIDS^39^ or Parkinson’s disease^40^.

Stronger correlations between cognitive subdomains were also observed in men compared to women at baseline and 3 months post-stroke. Based on our examination of the literature, there are no studies in stroke populations presenting sex-specific intercorrelations of cognitive domains. In a study in an elderly population, classifying the cognition scores for men resulted in fewer components compared to the same for women^41^. According to the authors of this study, this finding may suggest a more unified cognitive processing approach in men. Another extensive study reviewing sex-differences in cognition provided strong evidence for higher intercorrelations between cognition domains in men compared to women if the tests were presented in a more visuospatial manner similar to our results^42^. Conversely, women tend to demonstrate more variable interrelations across cognitive domains, which may stem from broader cognitive strategies or greater functional connectivity^43^.

Finally, for both sexes, this study found spurious associations between stroke volume or the extent of WMH and psychological status following stroke. However, previous publications have shown that WMH and stroke volume are strong predictors of cognitive and mood impairment^44,45^. Initially, we considered two explanations for this discrepancy: our FDR correction, or the use of age and stroke severity as control variables. However, the associations remained weak even without correction, and only mood scores—not cognitive scores—were correlated with the control variables. Further high-quality studies are required to conclusively determine the role of neuroradiological markers on these psychological outcomes.

Several limitations must be noted with respect to our findings. First, concerning the comparison between men and women, our modest sample size limits statistical power and increases the likelihood of Type II error when interpreting null findings. Therefore, the absence of significant sex differences in our results should not be interpreted as evidence that sex is unrelated to the post-stroke neurological or psychological outcomes of interest. Larger, sex-stratified samples will be necessary to quantify their magnitude and identify potential clinically meaningful sex-based differences. Moreover, the sample size restricted the number of variables analyzed to minimize the impact of multiple comparisons. Second, there was no data collected on the personal history of mood or anxiety disorders, one of the strongest predictors of post-stroke emotional impairments, which may limit our result’s generalizability. Third, knowing that stroke severity has been linked to post-stroke psychological status^2,5,24^, NIHSS score was taken as a numerical control variable in our analyses. However, the limited spectrum of stroke severity limits the generalizability of our results. Fourth, certain aspects of post-stroke status were not documented: substance use at follow-ups, complications such as pain and sleep disturbances, as well as social factors including isolation, family support, or discharge destination—all of which are known to influence psychological functioning, psychosocial recovery, and QoL^46–48^. Finally, the study setting did not allow the evaluation of gender-based differences^49^.

## Conclusions

This study highlights significant differences between men and women in the clinical presentation and post-stroke evolution. Since very limited research into post-stroke sex-stratified longitudinal inter-correlations between cognitive and socio-affective measures has been conducted, we believe our results are a notable contribution to the literature. It further emphasises the need for taking into account sex- and gender-specific factors in clinical evaluations for advancing personalized medicine and enhancing post-stroke recovery.

## Data Availability

The datasets generated and/or analysed during the current study are not publicly available due medical confidentiality but are available from the corresponding author on reasonable request.
